# Flexible ultrathin organic electrochemical transistor (OECT)-based portable system for monitoring and stimulation of bladder muscle

**DOI:** 10.1088/2058-8585/ae9bd1

**Published:** 2026-08-27

**Authors:** Minghui Zhu, Roshini Suresh, Bahareh Vahabi, Faezeh Arab Hassani

**Affiliations:** 1 School of Electrical, Electronic and Mechanical Engineering, University of Bristol, Bristol, United Kingdom; 2 School of Health and Social Wellbeing, University of the West of England, Bristol, United Kingdom; 3 School of Applied Sciences, University of the West of England, Bristol, United Kingdom

**Keywords:** flexible ultrathin organic electrochemical transistor array, bladder muscle, monitoring and stimulation, organ bath, force sensor

## Abstract

Patients with bladder underactivity experience difficulty in sensing bladder fullness and emptying the bladder efficiently, leading to chronic urinary retention. An implantable bidirectional device that combines capturing bladder smooth muscle activities with stimulation could enable closed-loop assistance and offer benefits compared to the common timed intermittent catheterization. Most existing bidirectional systems lean on passive metal electrodes as the interface, which provide no intrinsic signal amplification for low-amplitude bladder electromyography sensing and offer limited charge-injection capacity for effective and safe stimulation. Organic electrochemical transistors (OECTs) offer an attractive alternative with high sensitivity for biopotential measurements and effective stimulation capability due to their high-capacitance polymer channels. In this study, we used a custom-made portable system for a flexible ultrathin OECT array to shape a bidirectional recording and stimulation interface for bladder smooth muscle. We validate the system’s recording and stimulation performance under *ex vivo* conditions by using an organ bath setup equipped with a commercial force transducer as a mechanical reference. The array recordings showed stable baselines during inactive periods and clear and repeatable low-frequency output changes that matched force changes during spontaneous muscle contractions. The system also delivered programmed pulse trains that consistently triggered measurable contractions in the muscle, proving its reliable stimulation capability. These findings establish a practical foundation for OECT-enabled closed-loop bladder monitoring and stimulation and support the broader use of active organic bioelectronics as bidirectional interfaces for soft and dynamic organs.

## 1. Introduction

Bladder dysfunction influences a large population of people around the world, especially the elderly [1, 2]. Various related issues, including urinary incontinence, urinary frequency, and incomplete bladder emptying, significantly disrupt the quality of patients’ daily lives [3]. One of those conditions, underactive bladder, could lead to the loss of bladder fullness sensation, prolonged bladder voiding, or even failure to empty the bladder in typical periods [4, 5]. Current strategies for treating underactive bladder patients are to utilize intermittent catheterization at specific time intervals to remove urine from the bladder, which could lead to various complications such as urinary tract infection, urethritis, mental health problems, and a low quality of life [6–8].

Electromyographic (EMG) recording provides an overview of the electrical activity from the bladder muscle by monitoring its contraction and relaxation [9–12]. On the other hand, electrical stimulation could be employed as a method to induce muscle contraction to void the dysfunctional bladders [10, 12]. Passive electrodes have been used so far for both EMG recording and electrical stimulation of the bladder muscle to shape closed-loop therapies for bladder dysfunction [10, 12]. However, the passive electrodes are mostly able to detect EMG signals when the muscle experiences maximum activity, mainly during the bladder voiding phase [10–12]. Also, due to the low charge-injection capacity of passive electrodes, a high stimulation voltage/current is required to achieve effective muscle activation for bladder voiding [10, 12]. Organic electrochemical transistors (OECTs), as active organic devices, are capable of detecting small concentrations of key ions contributing to bladder muscle activities (i.e. Sodium (Na^+^), Potassium (K^+^), Calcium (Ca^2+^)) [13] and ion exchanges produced by tiny biopotential activities in the body [14, 15]. Previous studies have demonstrated OECTs’ potential for recording various electrophysiological activities, including electrocorticography [16], electrocardiography [17], and EMG [18]. The capability of ions penetrating into the organic semiconductor layer provides OECTs with gate-channel capacitances more than three orders of magnitude higher than those achieved with cutting-edge high-k dielectric materials [19]. As a result, higher charge injection capacity of OECTs allows a smaller voltage requirement for muscle stimulation that minimizes the risk of undesired electrochemical reactions, tissue damage, and electrode surface degradation [20–22].

Here, we use a flexible ultrathin OECT-based array, for both the recording of bladder muscle activity and its stimulation. The ultrathin OECT array sits conformably on the bladder muscle surface. Poly (3,4ethylenedioxythiophene) polystyrene sulfonate (PEDOT:PSS) channels, the most widely used OECT channel material [23], are used for the p-type depletion-mode OECTs in this work. We first demonstrate the *in vitro* characterization of the ultrathin OECT-based array by using a custom-made portable system and compare its performance against a parameter analyzer. Then, to validate the OECTs’ capability in recording and stimulation of the bladder muscle, we use an organ bath setting that measures the muscle activity by using a commercial force transducer as a reference. The commercial force transducer was used as a reference because it provides a direct and quantitative measurement of the mechanical contraction generated by the bladder tissue strip.

During recording, the OECT detects extracellular electrical-potential changes associated with detrusor activity, whereas the force transducer independently confirms that these electrical changes are accompanied by actual tissue contraction. At low force levels during isometric contractions, surface EMG amplitude generally exhibits a linear relationship with muscle force [24]. In addition, simultaneous electrical and mechanical measurements are physiologically relevant, spontaneous action potentials are confirmed to be fundamental processes for spontaneous excitation of detrusor smooth muscles [25]. During stimulation, the primary outcome of interest is whether electrical stimulation delivered through the OECT channel produces a real contraction of the detrusor tissue. The force transducer directly measures the magnitude and temporal profile of this evoked contraction and therefore provides an appropriate reference for evaluating stimulation effectiveness. The custom-made portable system for the array, allows easy *ex vivo* characterization of the system without the need for bulky measurement equipment. This work lays the groundwork for a future closed-loop platform that connects bladder activity monitoring to responsive stimulation.

## 2. Methods

### 2.1. Portable system overall design

**Figure 1.**
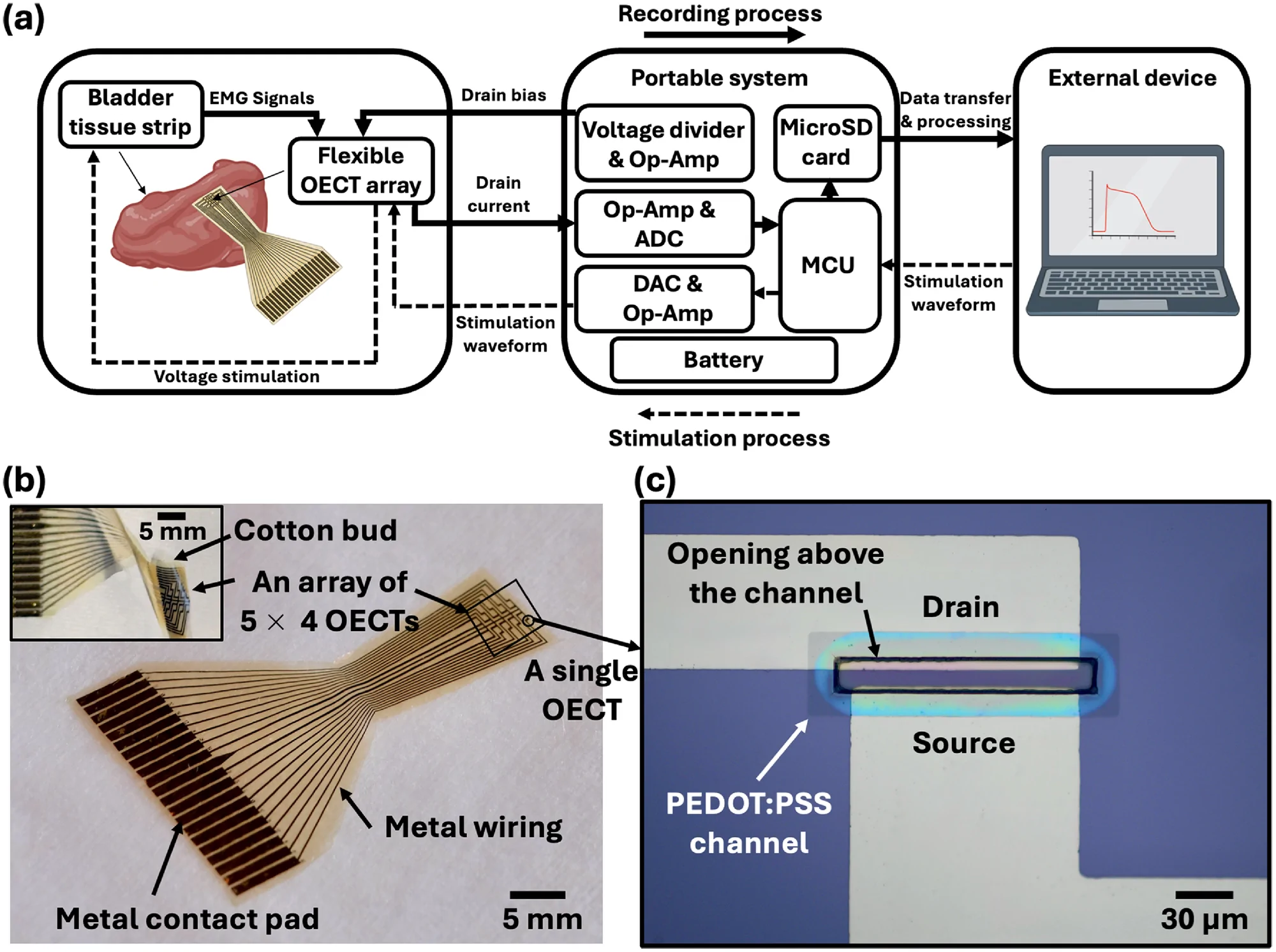
Overall bidirectional system for the OECT array. (a) Functional block diagram for the bidirectional system. (b) The image of the ultrathin OECT array. Inset: The photographic image of the array wrapped around a cotton bud’s tip. (c) A magnified microscopic image of a single OECT.

The OECT-based portable system consists of three parts: (i) A flexible ultrathin OECT array as a seamless interface with the tissue strip. (ii) A custom-made interface circuit (i.e. a data acquisition and control system) containing a long-lasting battery to provide the drain bias and stimulation waveform for the OECT array. (iii) An external device for processing the recording data, and also adjusting the magnitude and frequency of the stimulation waveform. An overview of the proposed system is shown in figure 1(a). During the recording process, the portable system provides a drain voltage bias to the OECTs and acquires biopotential signals from the tissue by measuring the OECTs’ drain current output. The drain current output was then amplified and converted to a voltage output via an analogue to digital (ADC) converter. The recorded data was transferred to the microcontroller unit (MCU) and then written to an external microSD card module and sent for processing to the external device. During the stimulation process, a stimulation pulse signal is applied by the portable system’s digital to analogue converter (DAC) to the drain and source pins of a selected OECT to electrically stimulate the tissue.

#### 2.1.1. Flexible ultrathin OECT array fabrication

The flexible ultrathin array includes 20 OECTs with 250 × 10 *μ*m^2^ PEDOT:PSS channels with a uniform thickness of 450 nm. Figure 1(b) shows the array of 5 × 4 OECTs with a total area of 11.8 mm^2^ (marked within a square), connecting wires, and contact pads. The flexibility of the device is illustrated by wrapping it around a cotton bud’s tip (Inset to figure 1(b)). The OECTs share the same drain contact pads and 20 individual source contact pads for connecting to the portable system. This wiring design is used to reduce the number of wiring and contact pads for easier connection to the circuit and minimize unnecessary space occupation in the organ bath during *ex vivo* testing. A magnified microscopic image of a single OECT is shown in figure 1(c).

The steps of the fabrication process for the OECT array were reported in our previous work [26] and are presented in figure S1. The fabrication starts by depositing a 300 nm-thick silicon oxide (SiO_2_) layer on a silicon (Si) carrier wafer (figure S1(a)) and then spin-coating a 1.5 *µ*m-thick PI substrate (figure S1(b)). Chromium (Cr)/gold (Au) contact pads were patterned by photolithography, metal deposition, and lift-off (figures S1(c)-(g)). PEDOT:PSS and S1813 photoresist were subsequently spin-coated, patterned and etched to define the channel (figure S1(h)–(l)). This step is then followed by deposition and patterning of an SU-8 photoresist layer as an encapsulation layer that leaves the channel area exposed (figures S1 (m)–(o)). Finally, the device was peeled off from the carrier wafer to obtain a 5 *µ*m-thick flexible OECT array (figure S1 (p)). Peeling off the array from the carrier wafer is shown in figure S2.

#### 2.1.2. Interface circuit design

**Figure 2.**
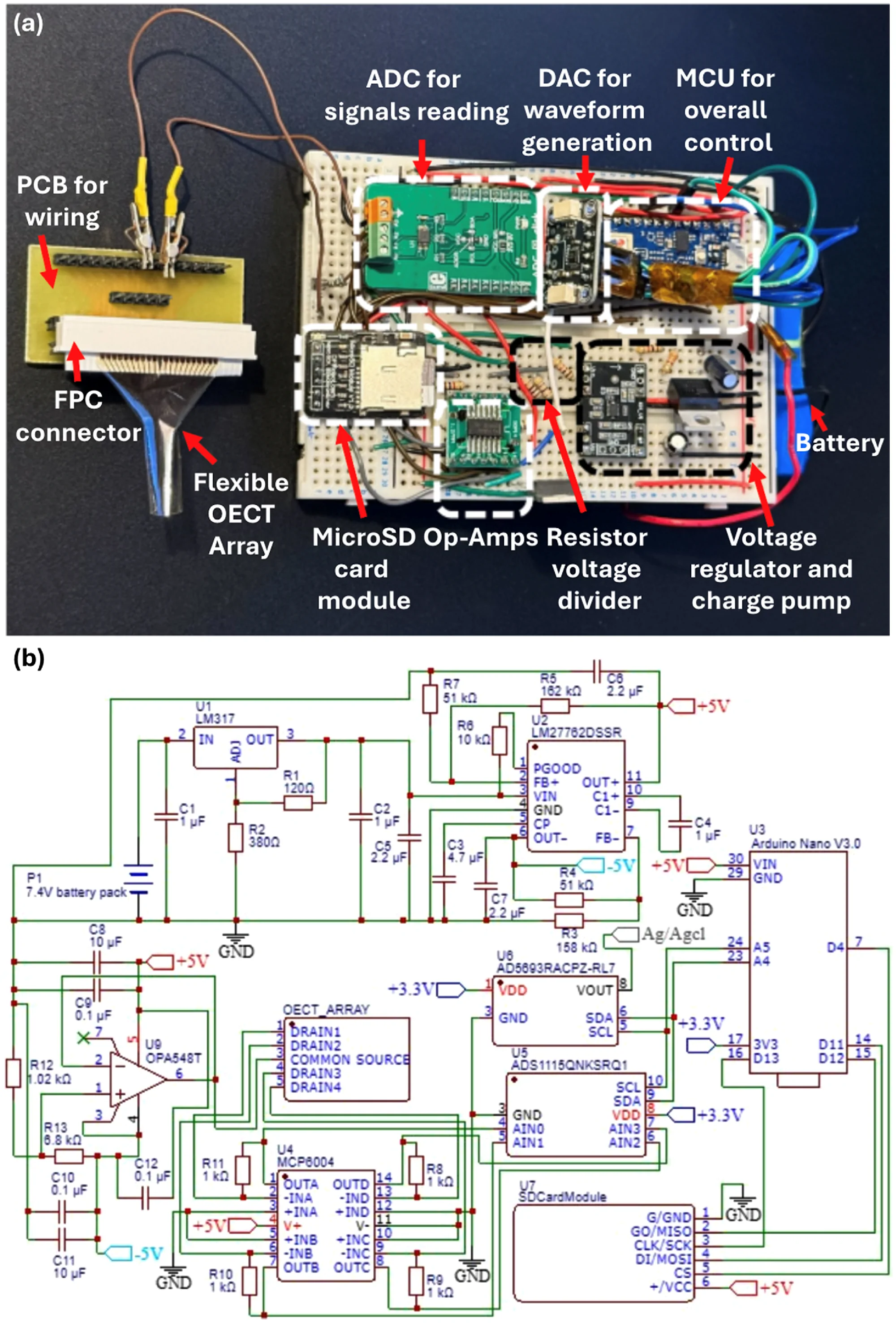
Portable system design. (a) Photograph of the portable system connected to the OECT array. (b) Circuit schematic of the interface and the OECT array.

A +7.4 V power supply from a lithium-ion rechargeable battery pack is passed through an LM317 low-dropout voltage regulator, and an LM27762 low-noise positive and negative output integrated charge pump to achieve a stable ±5 V power supply for the overall system. The MCU of an Arduino Nano controls the AD5693R, 16-bit low-drift DAC, via an inter-integrated circuit (I^2^C) bus to generate different testing pulses, including the gate voltage *V*_G_, or the square wave used for the electrical stimulation. An OPA192 operational amplifier (Op-Amp) acts as a voltage buffer between the DAC and the target OECT terminal to increase the current-driving capacity of the supply voltage. The output of the AD5693R DAC is connected to the gate terminal of the OECT via an external silver/silver chloride (Ag/AgCl) probe for DC characterization. An OPA548T Op-Amp acts as a voltage buffer between the voltage divider circuit and the target OECT terminal for providing the constant drain voltage *V*_D_ to the target OECT terminal. The *I*_D_ of each OECT is routed through the corresponding source terminal to a separate channel of the MCP6004 Op-Amp, where it is converted into the amplified output voltage (*V*_OUT_) via the transimpedance configuration. Here, the feedback resistors of the Op-Amps (*R*_f_) are set to 1 kΩ to achieve a gain of 1000, matching the input range of the ADS1115 ADC, with a 16-bit resolution and a sampling rate of up to 860 samples per second. The ADS1115 was configured with a ±4.096 V full-scale input range, corresponding to a readout resolution of approximately 125 *µ*V per ADC count. Owing to the processing speed of the MCU used in the current system, outputs from 4 of the 20 channels could be recorded and processed simultaneously while maintaining the desired sampling rate. A photograph of the portable system circuit connected to the OECT array and the circuit schematic are presented in figures 2(a) and (b), respectively. The photo of the backside of the breadboard that housed the battery pack is presented in figure S4.

### 2.2. *In vitro* setup

**Figure 3.**
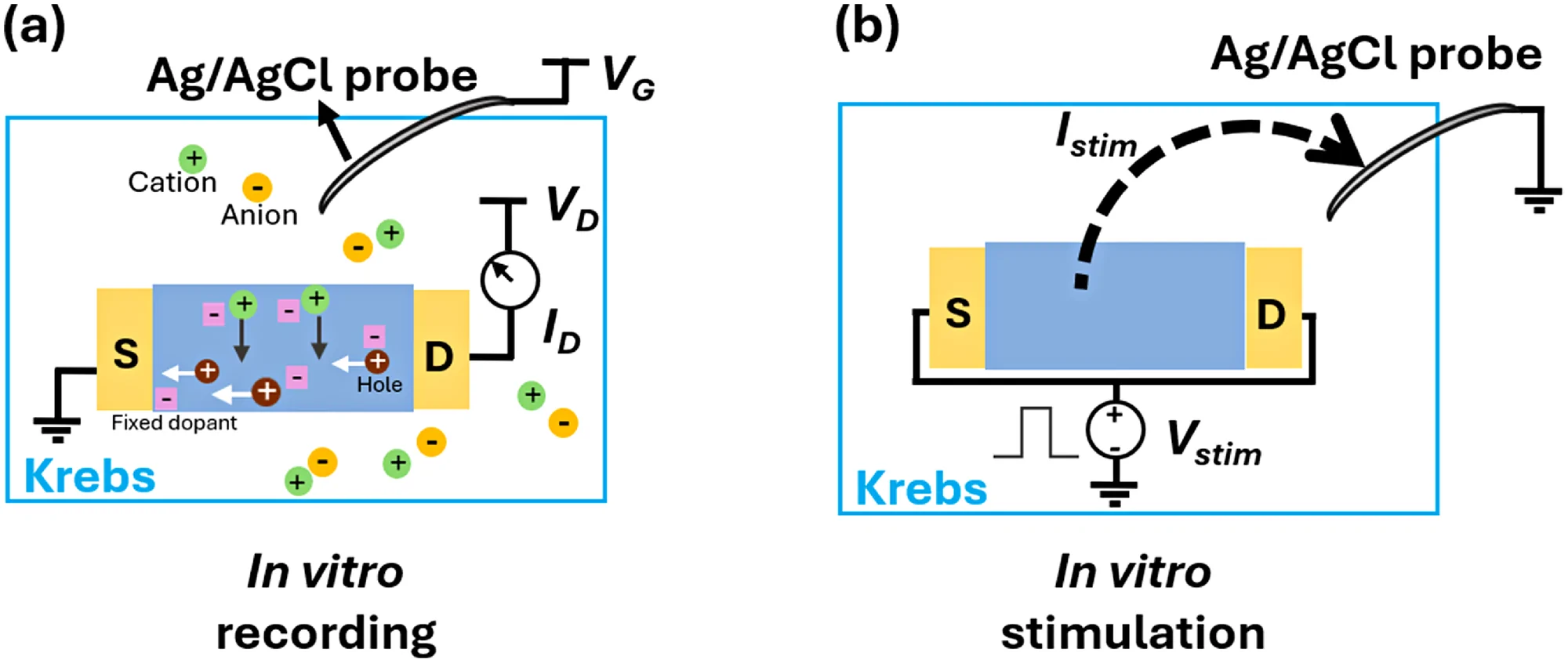
Schematics of the *in vitro* setup for an OECT during recording and stimulation. (a) The configuration during the recording period. (b) The configuration during the stimulation period.

The OECT array was characterized using a Keithley 4200 A-SCS parameter analyzer and the custom-built portable system to benchmark its performance for both recording and stimulation. The schematic of the *in vitro* setup for an OECT during recording and stimulation is presented in figures 3(a) and (b), respectively. Krebs–Henseleit solution was used as the electrolyte in all tests. In figure 3(a), the drain voltage *V_D_* was provided by the portable system. The applied gate voltage (*V*_G_) pushes mobile cations from the Krebs solution into the OECT’s PEDOT:PSS channel that compensate the fixed sulfonate anions of PSS^-^. This process results in de-doping of the channel (i.e. reducing PEDOT^+^) from holes as the majority carriers and therefore modifies its conductivity **[**14**]**. P-type depletion-mode OECTs were used here due to their rapid initial responses, whereas using accumulation-mode OECTs in the future could reduce power consumption for chronic applications **[**27**]**. The output drain current *I*_D_ measured by the parameter analyzer was compared with the amplified output voltage *V*_OUT_ recorded by the portable system during the output, transfer, and transient characterization measurements. For the stimulation in figure 3(b), the source and drain terminals of the selected OECT are connected together so that the exposed PEDOT:PSS channel functions as a stimulation electrode. A stimulation waveform *V*_stim_ was applied separately using the parameter analyzer and the portable system between the channel and the grounded Ag/AgCl (i.e. the current sink). This generated a stimulation current *I*_stim_ through the Krebs solution. The resulting *I*_stim_ was measured using the parameter analyzer for both systems to compare their performance.

### 2.3. *Ex vivo* setup

#### 2.3.1. Materials preparation

**Figure 4.**
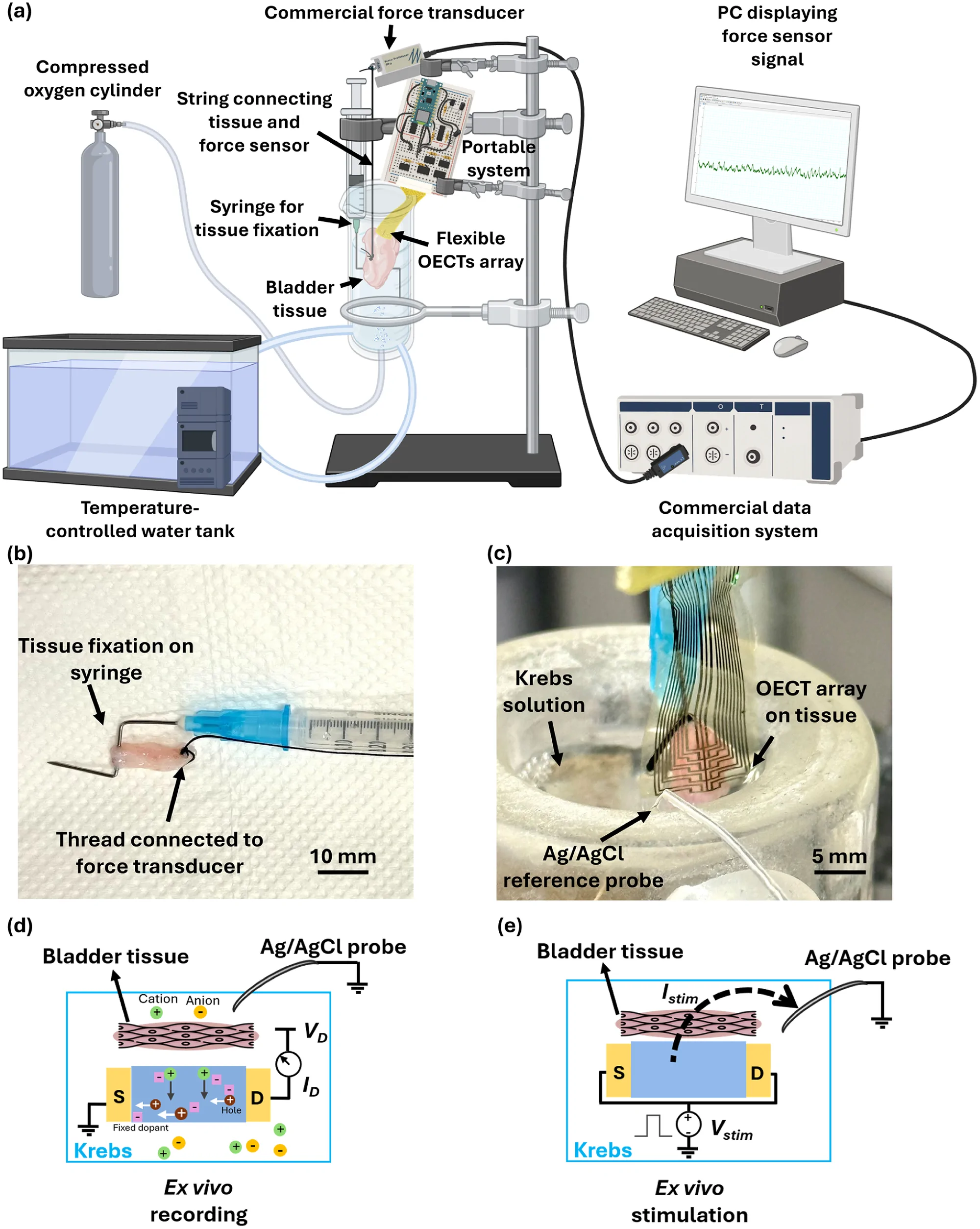
Schematics of the overall setup for the *ex vivo* experiments. (a) The image of the components supported by the laboratory stand including the organ bath, the syringe, the OECT array and portable system board, and the force transducer, the commercial data acquisition unit connected to a PC, the temperature-controlled water tank, and the oxygen cylinder. (b) Tissue strip fixation using a syringe needle and a thread. (c) The OECT array placement on tissue and an Ag/AgCl reference electrode inserted inside the Krebs solution. (d) The configuration during the recording period. (e) The configuration during the stimulation period.

The *ex vivo* setup [28, 29] consisted of a double-walled organ bath filled with Krebs–Henseleit (Krebs) solution, a syringe needle to secure the prepared tissue strip, and a commercial force transducer (Teaching Force Transducers MLTF050, AD Instruments) connected to the tissue via a thread to detect tissue contractions. The OECT array was then positioned on the tissue strip and connected to the portable system to monitor the tissue activity alongside the force transducer. The applied force from tissue to the force transducer induces changes in its resistance, producing a corresponding change in the output voltage. The transducer can measure isometric forces from 0 to 50 g and is connected to a commercial data acquisition unit (PowerLab 26, AD Instruments). The force sensor output voltage data were then transferred to a PC for real-time display and recording. The schematic of the complete *ex vivo* experimental setup is presented in figure 4(a).

Krebs solution was freshly prepared according to the following composition: 5.0 l Deionized (DI) water was added to a clean bottle and stirred continuously. 34.6 g Sodium Chloride (NaCl), 10.5 g D-Glucose, 10.5 g Sodium Hydrogen Carbonate (NaHCO_3_), 1.7 g Potassium Chloride (KCl), 1.45 g Magnesium Sulfate (MgSO_4_), 0.8 g Monopotassium Phosphate (KH_2_PO_4_), 9.5 ml 1 M Calcium Chloride (CaCl_2_) were added sequentially and allowed to dissolve completely.

The outer wall of the organ bath was connected to a temperature-controlled water circulator to maintain the temperature of the Krebs solution at 37 °C, while a compressed oxygen cylinder was connected to the chamber inlet to provide continuous gas bubbling for solution oxygenation. The cylinder’s valve regulated the gas flow to ensure that only a gentle stream of bubbles was present, minimizing mechanical disturbances that could affect the OECT array and force sensor readouts. Temperature control for the *ex vivo* experiment was conducted by using a digital thermostat (Grant Optima, Grant Instruments) and a water tank, with a stability of ± 0.05 °C. The thermostat includes an integrated pump for external circulation and was connected to the outer layer of the organ bath.

For preparing the tissue strip, fresh female pig bladders were obtained from a local abattoir and kept refrigerated. The whole bladder was first cut longitudinally from the middle part of the posterior wall with fine scissors (figure S3(a)). The bladder was then unfolded to show the inner surface. An approximately 15 mm × 5 mm, 0.2 g-tissue specimen containing all layers (urothelium, submucosal, detrusor and adventitia) was then extracted from the whole expanded bladder tissue (figure S3(b)). One end of the tissue strip was then pierced by a stainless-steel syringe needle to secure, while the opposite end was tied with a non-elastic thread that was later connected to the hook of the commercial force sensor (figure 4(b)). The tissue strip was partially immersed in the solution, and the flexible array was placed on the tissue surface near the immersed region. The ultrathin and flexible structure of the array allowed it to adhere to the tissue and remain in place without additional support. The array was connected to the custom portable system mounted on the stand. An Ag/AgCl electrode was immersed in the Krebs solution close to the tissue strip and connected to the portable system’s ground, providing a stable reference potential for readout and reducing the baseline drift during recording. In future *in vivo* testing, the Ag/AgCl electrode could be replaced by a surface electrode on the skin or a subdermal needle to provide the reference. A magnified view of the OECT array and the Ag/AgCl electrode positioning during the experiment is shown in figure 4(c). The schematic of the *ex vivo* setup for an OECT during recording is presented in figure 4(d). *V_D_* was provided by the portable system. During spontaneous contraction, transmembrane ionic currents in the detrusor smooth muscle cells generate potential variations (i.e. *V*_G_) in the Krebs solution. The grounded Ag/AgCl electrode defines the bath reference potential. The electrical potential modulation between the grounded Ag/AgCl reference electrode (RE) and muscle pushes mobile cations from the Krebs solution into the OECT’s PEDOT:PSS channel that compensate the fixed sulfonate anions of PSS^-^. This process results in de-doping of the channel (i.e. reducing PEDOT^+^) from holes as the majority carriers and therefore modifies its conductivity. Figure 4(e) shows the schematic configuration during the stimulation period. The source and drain terminals of the selected OECT are connected together so that the exposed PEDOT:PSS channel functions as a stimulation electrode. *V*_stim_ is applied by the portable system between the channel and the grounded Ag/AgCl (i.e. the current sink), producing a stimulation current *I*_stim_ through the Krebs solution. The resulting electric field could depolarize the detrusor smooth muscle cells, causing the exchange of ions between the cells and the Krebs solution, and activate the contraction of the tissue strip.

#### 2.3.2. Tissue equilibration and activity periods

**Figure 5.**
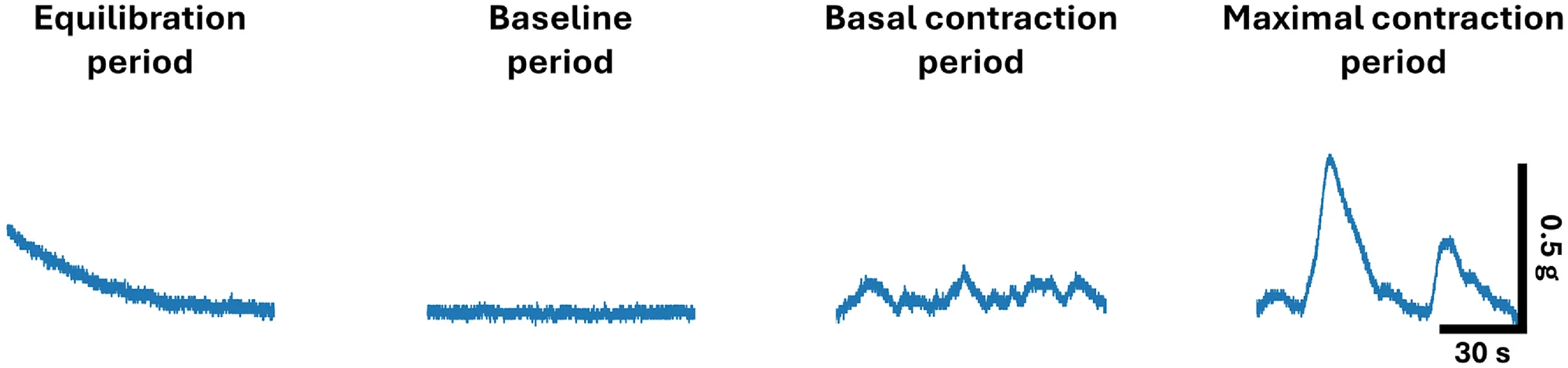
Representative tissue responses periods defined by the force transducer output.

The force transducer records the tissue contractions continuously during the experiment with a sample rate of 40 Hz. The tissue strip tied to the force transducer was allowed to equilibrate for approximately 10 min before starting to record the baseline. The smooth muscle often shows transient hypercontractility or suppression after dissection and mounting, which needs time to settle metabolism, ion gradients, and receptor coupling. The equilibration period ensures that bath conditions (e.g. a fixed temperature of 37 °C and continuous gassing with oxygen (O_2_) to maintain oxygenation and pH buffering) are steady prior to recording the responses. Also, the equilibration provides time to adjust the applied preload force to the tissue by the force transducer, which allows passive tension to stabilize and improve the baseline stability. Following the equilibration, the preparation generally includes a baseline period of approximately 20 min where no tissue response is visible. Spontaneous contractions then became detectable and increased over the subsequent 10 min (Basal contraction period), reaching a maximum responding stage (Maximal contraction period). This maximal activity could persist for several hours when bath conditions were maintained within the physiological range. Figure 5 presents baseline, basal and maximal contraction period for a testing tissue strip. The duration of each stage varied between different testing tissues.

#### 2.3.3. Portable system setting

During the array recording, the portable system was continuously providing a *V*_D_ of −0.65 V to the selected OECT channel. The selected channel was connected to the related data acquisition pin of the portable system. Manual operation was required to start each recording period on the portable system. When a recording on the portable system was initiated, a corresponding timestamp marker was added to the force transducer data stream to enable subsequent alignment between the force and OECT recordings. Between each recording period by the portable system, a 5 min buffer period was introduced to allow the microcontroller to transfer data from its on-chip memory to an external microSD card. The recorded files were then transferred to a PC for further analysis and archiving. Multiple recordings were acquired during the maximum responding period of the tissue to confirm the repeatability of the responses.

During the stimulation process, a selected OECT channel delivered a 1 V, 50 Hz square waveform with 5 s duration and a 1-millisecond pulse width to the tissue strip. An Ag/AgCl electrode connected to system ground served as the current sink electrode through the electrolyte. In future *in vivo* testing, the Ag/AgCl electrode could be replaced by a patterned gold electrode coated with a PEDOT:PSS layer that is positioned near the flexible OECT array. Stimulation was initiated via the microcontroller, and a timestamp marker was simultaneously added to the force transducer stream at the start of stimulation, enabling alignment of the tissue mechanical response with the applied stimulation.

#### 2.3.4. Overall experimental schedule

The full set of results was obtained across five separate experimental sessions, each using a different testing tissue strip. Specifically, Experiment 1 completed recording measurements on Tissue 1 using Array 1. Experiment 2 conducted recording tests on Tissue 2 using Array 2. Experiment 3 repeated combined recording and stimulation tests on Tissue 3, again using Array 1. Experiment 4 performed electrical stimulation on Tissue 4 using Array 3. Finally, Experiment 5 carried out continuous stimulation testing on Tissue 5 using Array 4. The overview of the experimental schedule is presented in table 1. This multi-session design ensured consistent data collection and enabled evaluation of the stability of the experimental conditions.

**Table 1.** Overview of the experimental schedule.

Experiment	Tissue	Array	Result
1	$Tissue{\text{ }}1$	$Array{\text{ }}1$	Recording
2	$Tissue{\text{ }}2$	$Array{\text{ }}2$	Recording
3	$Tissue{\text{ }}3$	$Array{\text{ }}1$	Recording&Stimulation
4	$Tissue{\text{ }}4$	$Array{\text{ }}3$	Stimulation
5	$Tissue{\text{ }}5$	$Array{\text{ }}4$	Stimulation

## 3. Results

### 3.1. *In vitro* characterization

**Figure 6.**
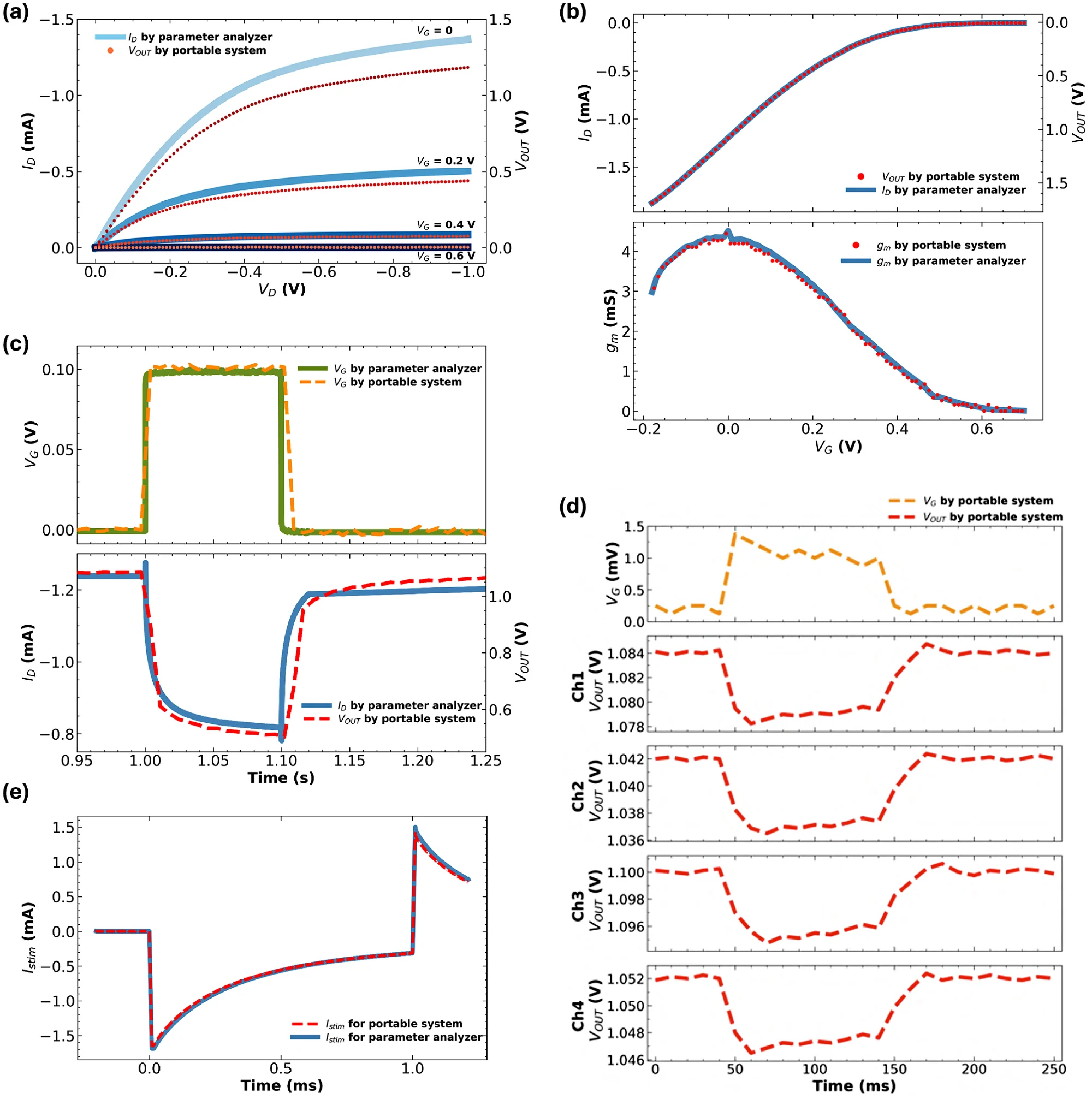
The OECT *in vitro* characterization with both the commercial parameter analyzer and the portable system. (a) The output characteristics for various *V*_G_. (b) The transfer characteristics at *V*_D_= −0.65 V. (c) The transient characteristics at *V*_D_ = −0.65 V. (d) Multi-channel transient characteristics at *V*_D_ = −0.65 V. (e) The stimulation current response measurements in response to a 1 V, 100 Hz square waveform voltage applied to the connected drain and source terminals.

For the output characteristics in figure 6(a), *V*_D_ was swept from −1 V to 0 V while *V*_G_ increased from 0 to 0.6 V in 0.2 V increments. Both systems produced the expected depletion-mode PEDOT:PSS OECT behavior, with *I*_D_ and *V*_OUT_ decreasing under positive *V*_G_ due to ionic de-doping and showing a linear-to-saturation transition as *V*_D_ increases beyond the pinch-off point [16]. The measured *V*_OUT_ was slightly lower than the expected amplification of 1000 due to contact and interconnection resistances in the readout circuit. Transfer characteristics in figure 6(b) were measured at *V*_D_ = −0.65 V with *V*_G_ swept from −0.2 V to 0.7 V. The two platforms showed similar *I*_D_—*V*_G_ and *g*_m_ characteristics, including a *g*_m_ peak value of ≈4.5 mS at *V*_G_ = 0. The transient characteristics from both systems (figure 6(c)) were assessed by applying a 100 mV, 1 Hz *V*_G_ square wave with *V*_D_ = −0.65 V. Both systems reported similar output response trends, with the *V*_OUT_ again being slightly lower than the expected amplification value, while maintaining similar ON to OFF response times. To evaluate the consistency of transient characteristics between various OECTs, we performed multi-channel recording for 4 neighboring OECTs (i.e. Channels (Ch) 1–4) of the array simultaneously. For this experiment, a low-amplitude gate-voltage pulse *V*_G_ was generated by the portable system (1 mV amplitude, 1 Hz frequency, 100 ms pulse width), chosen to approximate the electrophysiological signal level expected in the *ex vivo* experiments. As shown in figure 6(d), all four OECTs exhibited clear and repeatable decreases in *V*_OUT_ upon *V*_G_ application, followed by recovery after the pulse returned to the baseline. The steady-state baselines differed slightly among devices, reflecting normal inter-device variability. In response to the *V*_G_ signal, the four channels exhibited comparable *V*_OUT_ amplitudes of 5.75, 5.50, 5.25, and 5.48 mV for Ch 1–4 in the sample, respectively. These results indicate that no obvious crosstalk was detected between the transistors under the tested conditions. Finally, stimulation capability was validated by delivering a 1 V, 100 Hz square waveform voltage to the connected drain and source terminals and measuring the current *I*_G_ between the OECT channel and an Ag/AgCl electrode current sink. The resulting current responses show strong agreement with similar peak and decay levels (figure 6(e)), demonstrating that the custom platform can accurately generate and deliver stimulation waveforms comparable to the parameter analyzer.

**Figure 7.**
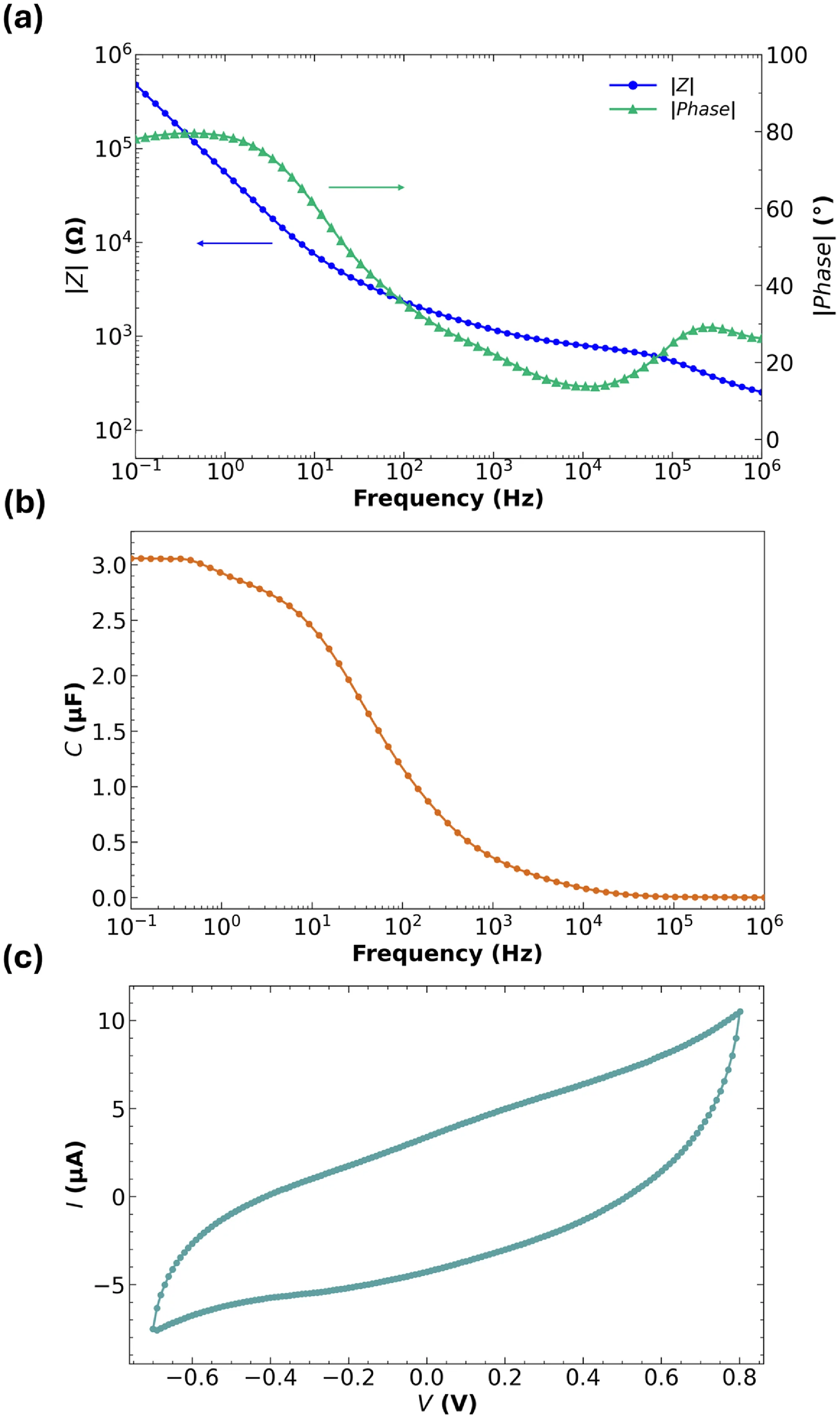
Characterization of the OECT channel. (a) Electrochemical impedance spectroscopy impedance modulus and phase vs frequency. (b) Electrochemical impedance spectroscopy capacitance vs frequency. (c) Cyclic voltammogram.

A PalmSens Nexus equipment was used to conduct the electrochemical impedance spectroscopy (EIS) and cyclic voltammetry (CV) tests for extracting the OECT channel’s capacitance. A three-electrode setup was used as the configuration for the measurements, with the OECT channel as a working electrode (WE), an Ag/AgCl electrode as a RE, and a platinum wire electrode as a counter electrode. For the EIS measurement, a small AC modulation of 50 mV with a frequency range of 0.1 Hz to 1 MHz was applied to the WE with respect to the RE. The impedance modulus |*Z*| and phase versus frequency curves are shown in figure 7(a). The |*Z*| response exhibits a capacitance-dominated region at low frequencies and a more resistive behavior at high frequencies. A similar trend can also be observed from the phase curve, where the phase angle approaches 90° at low frequencies, indicating ideal capacitive behavior, and in high frequencies, the values are gradually shifting towards 0°, corresponding to more resistive behavior. The capacitance of the OECT channel at different frequencies (figure 7(b)) was calculated by the following equation from the EIS data [30]:

\begin{equation*}C = \frac{1}{{2\pi \times f \times \left| {{Z^{{\mathrm{img}}}}} \right|}}\end{equation*}

where ${Z^{{\mathrm{img}}}}$ is the imaginary part of the impedance and f is the frequency. The calculated capacitance exhibits a plateau in the low-frequency range. By averaging the capacitance values between 0.1 Hz and 10 Hz, an approximate capacitance of 2.88 *µ*F was obtained. The capacitance of the OECT channel was also evaluated by fitting the EIS result with an equivalent Randles circuit. The Randles circuit consisted of a resistor and a capacitor in parallel, representing the polymer film resistance and capacitance, respectively, in series with a resistor representing the electrolyte resistance [31]. A capacitance of 2.75 *µ*F was extracted from the equivalent circuit, which is in good agreement with the value calculated directly from the EIS data.

The CV testing was performed with a scan rate of 0.5 V s^−1^ and the voltage was swept between −0.7 V and 0.8 V, as shown in figure 7(c). The CV curves showed a broad capacitive-like response without distinct redox peaks, indicating electrochemical charging of the PEDOT:PSS channel. The capacitance of the OECT channel was calculated using the following equation [32]:

\begin{equation*} {C = \frac{{{{\mathop \smallint \nolimits}}\left| I \right|\left| {{\mathrm{d}}V} \right|}}{{2\pi \times v \times \left( {{V_{{\mathrm{max}}}} - {V_{{\mathrm{min}}}}} \right)}}} \end{equation*}

where I is the measured CV current, v is the scan rate, and ${V_{{\mathrm{max}}}} - {V_{{\mathrm{min}}}}$ corresponds to the voltammogram potential window. A similar capacitance value of 2.86 *µ*F was obtained from the CV data, which closely matches the capacitance value extracted from the EIS measurement.

### 3.2. *Ex vivo* recording

#### 3.2.1. Multi-channel recording

**Figure 8.**
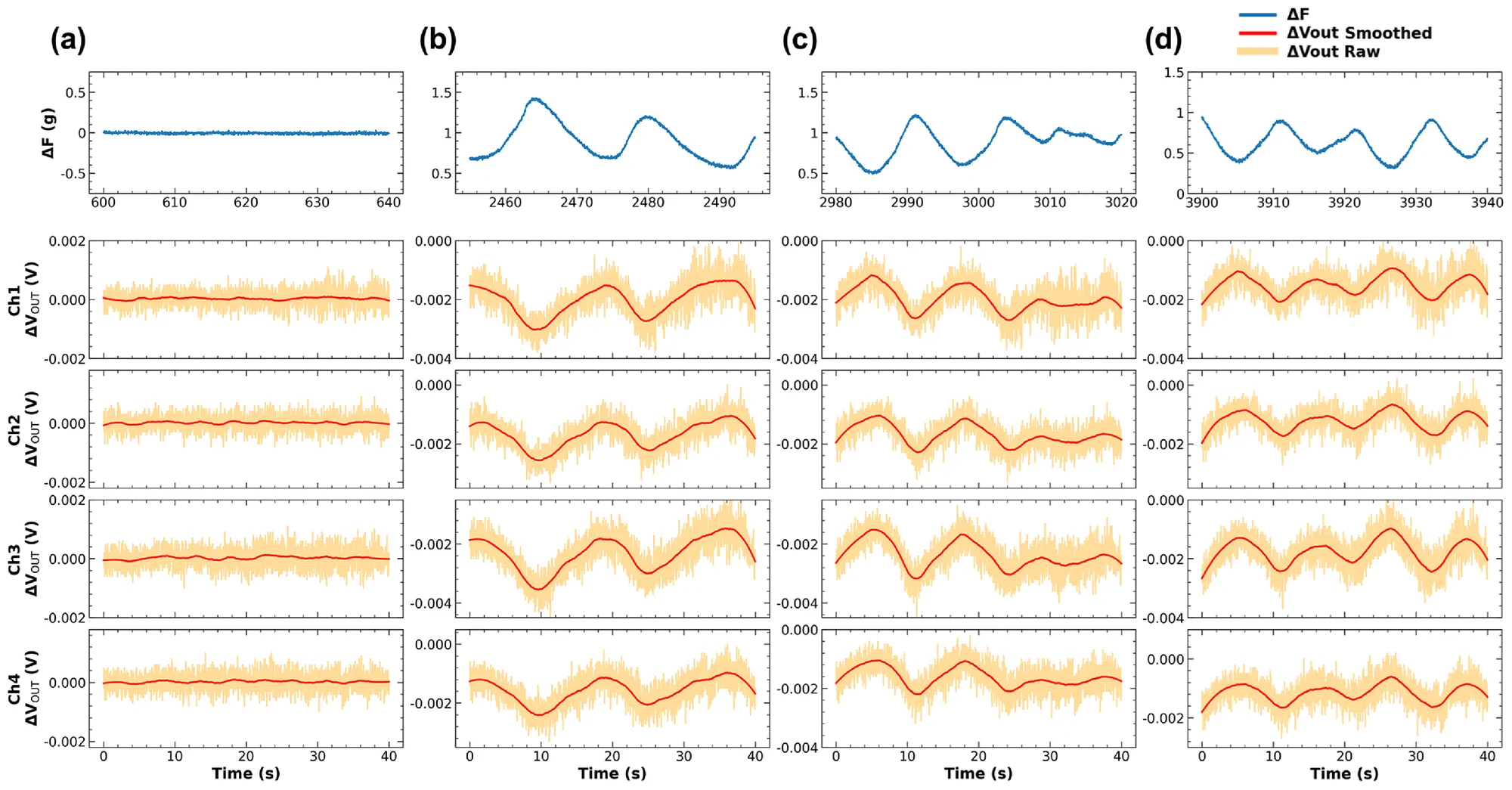
Simultaneous force transducer output and multi-channel recordings for 4 channels of the OECT array 1 on the tissue strip 1. (a) Baseline period recording. (b–d) Maximal contractions period recordings during three separate 40 s-blocks.

Multi-channel recording of the tissue strip was performed by measuring 4 OECTs (i.e. Ch 1–4) of the array simultaneously against the force transducer signal (Experiment 1). Current hardware constraints limit the number of channels that can be used at once for continuous recording. The output voltages for the tested OECT array 1 on tissue strip 1 are presented together with the force transducer signal in figure 8. For each channel, the recording is conducted during four 40 s-block. The tissue is in the baseline period during the first 40 s-block and in the maximal contraction period during the subsequent three blocks. The tissue activity was recorded during three separate 40 s-blocks to investigate the repeatability of responses. Raw channel traces were overlaid with Savitzky–Golay–smoothed curves to highlight low-frequency trends while preserving the local maxima and minima during the recording. ${ }{F_{{\text{ baseline}}}}$ and ${ }{V_{{\text{OUT baseline}}}}$ are the mean values for the smoothed force and the channel output voltage, respectively, for the tissue at the baseline period. The force output ${{\Delta }}F{\text{ }}$ was expressed as the value of the force transducer $F\left( t \right)$ deviation from baseline ${F_{{\text{ baseline}}}}$ with the unit of grams. The portable-system readout ${{\Delta }}{V_{{\mathrm{OUT}}}}$ was defined as changes in the smoothed channel output ${ }{V_{{\mathrm{OUT}}}}\left( t \right)$ relative to its baseline value ${ }{V_{{\text{OUT baseline}}}}$. The baseline noise ${\sigma _{{\mathrm{baseline}}}}{ }$ was quantified as the standard deviation of ${{\Delta }}{V_{{\mathrm{OUT}}}}$ using the following equation:

\begin{equation*}\begin{array}{*{20}{c}} {{\sigma _{{\mathrm{baseline}}}} = \sqrt {\frac{1}{{N - 1}}\mathop \sum \limits_{i = 1}^N {{\left( {{ }{V_{{\mathrm{OUT}}}}\left( t \right) - { }{V_{{\text{OUT baseline}}}}} \right)}^2}} } \end{array}\end{equation*}

where *N* is the number of the data points during the recording period. The recording quality is evaluated using the signal-to-noise ratio ${\mathrm{SN}}{{\mathrm{R}}_{{\mathrm{dB}}}}$, which relates the maximum signal deflection during tissue activity to the baseline noise level, as given by the following equation:

\begin{equation*}\begin{array}{*{20}{c}} {{\mathrm{SN}}{{\mathrm{R}}_{{\mathrm{dB}}}}{\text{ = }}20{\mathrm{lo}}{{\mathrm{g}}_{10}}\left( {\frac{{{{\Delta }}{V_{{\mathrm{OUT,max}}}}}}{{{\sigma _{{\mathrm{baseline}}}}}}} \right)} \end{array}\end{equation*}

where ${{\Delta }}{V_{{\mathrm{OUT,max}}}}$ is the largest absolute deflection of the ${{\Delta }}{V_{{\mathrm{OUT}}}}$ from the ${ }{V_{{\text{OUT baseline}}}}$ during the tissue activity period. The peak-to-peak sensitivity *S*_pp_ is used to assess recording sensitivity by quantifying the peak-to-peak portable system response relative to the corresponding peak-to-peak force transducer variation:

\begin{equation*}\begin{array}{*{20}{c}} {{S_{{\mathrm{pp}}}} = - \frac{{{{\Delta }}{V_{{\text{OUT, pp}}}}}}{{{{\Delta }}{F_{{\mathrm{pp}}}}}}{ }} \end{array}\end{equation*}

where ${{\Delta }}{V_{{\text{OUT, pp}}}} = \Delta {V_{{\text{OUT, max}}}} - {{\Delta }}{V_{{\text{OUT, min}}}}$ was obtained from the Savitzky–Golay smoothed ${{\Delta }}{V_{{\mathrm{OUT}}}}$ trace and ${{\Delta }}{F_{{\mathrm{pp}}}} = \Delta{F_{{\text{ max}}}} - {{\Delta }}{F_{{\text{ min}}}}$ from the simultaneously recorded force deviation within the responding window.

During the baseline period recording (figure 8(a)), the force trace indicated negligible contractile activity, and the ${{\Delta }}{V_{{\mathrm{OUT}}}}$ traces of all channels remained close to baseline, showing only small fluctuations with minimal drift. The baseline noise ${\sigma _{{\mathrm{baseline}}}}$ was calculated using equation (3) and was tightly grouped across the four channels, with values of 0.03370, 0.03310, 0.04785, and 0.03583 mV for Ch1 to Ch4, respectively. These results indicate a post-processed statistical measure of baseline fluctuations and good inter-channel consistency in the absence of tissue activity, which provides a stable reference for the active-state recordings in the subsequent blocks.

During tissue activity (figures 8(b)–(d)) the array exhibited clear low-frequency deflections in ${{\Delta }}{V_{OUT}}$ that tracked changes in ${{\Delta }}F$ with the expected polarity for the depletion-mode OECTs: As the tissue strip contracts, the force transducer output increases, and the tissue generates an increasing biopotential. The increasing biopotential enables the injection of cations from the electrolyte into the OECT channel and depleting the channel from carriers (de-doping). This process decreases the drain current and the corresponding portable-system output, resulting in lower ${{\Delta }}{V_{{\mathrm{OUT}}}}$ values. Therefore, the dip shown in the ${{\Delta }}{V_{{\mathrm{OUT}}}}$ trace coincided with the maxima in ${{\Delta }}F$ during tissue contraction, whereas the peak of the ${{\Delta }}{V_{{\mathrm{OUT}}}}$ occurred during tissue relaxation, when ${{\Delta }}F$ returned to its minimum.

${{\Delta }}F{\text{ }}$ in figure 8(b) shows two pronounced contractions and two relaxation peaks for the tissue strip. The portable system readout for Ch1 displays two corresponding dips and peaks in ${{\Delta }}{V_{{\mathrm{OUT}}}}$, with ${\mathrm{SN}}{{\mathrm{R}}_{{\mathrm{dB}}}}$ = 39.07 dB and ${S_{{\mathrm{pp}}}}$ = −1.92 mV g^−1^. Results from Ch2 follow the same pattern, with ${\mathrm{SN}}{{\mathrm{R}}_{{\mathrm{dB}}}}$ = 37.78 dB and ${S_{{\mathrm{pp}}}}$ = −1.74 mV g^−1^. Ch3 and Ch4 show a similar pattern for ${{\Delta }}{V_{{\mathrm{OUT}}}}$ with ${S_{{\mathrm{pp}}}}$ = −2.38 mV g^−1^, ${\mathrm{SN}}{{\mathrm{R}}_{{\mathrm{dB}}}}$ = 37.40 dB and ${S_{{\mathrm{pp}}}}$ = −1.66 mV g^−1^, ${\mathrm{SN}}{{\mathrm{R}}_{{\mathrm{dB}}}}$ = 36.58 dB, respectively. ${{\Delta }}F$ shown in figure 8(c) exhibited more frequent oscillatory activity, indicating repeated contractions within the 40 s window. ${{\Delta }}{V_{{\mathrm{OUT}}}}$ of the four OECT channels tracked these repeated force variations clearly, with ${\mathrm{SN}}{{\mathrm{R}}_{{\mathrm{dB}}}}$ values of 35.77–38.10 dB and ${S_{{\mathrm{pp}}}}$ values of −1.65 to −2.44 mV g^−1^. Ch3 exhibited the largest absolute peak-to-peak response sensitivity, while Ch4 showed the smallest peak-to-peak response. Similar results occur in figure 8(d) with a more frequent contraction pattern. ${{\Delta }}{V_{{\mathrm{OUT}}}}$ curves of all the channels follow the reverse patterns of ${{\Delta }}F$ signal clearly, with ${\mathrm{SN}}{{\mathrm{R}}_{{\mathrm{dB}}}}$ range of 33.28–35.81 dB and ${S_{{\mathrm{pp}}}}$ range of −1.77 to −2.42 mV g^−1^. The relative channel performance was unchanged, with Ch3 remaining the most sensitive channel and Ch4 the least sensitive. ${\sigma _{{\mathrm{baseline}}}}$, $\overline {{\mathrm{SN}}{{\mathrm{R}}_{{\mathrm{dB}}}}} $ (i.e. the mean ${\mathrm{SN}}{{\mathrm{R}}_{{\mathrm{dB}}}}$ value across the three responding recordings), and ${\bar S_{{\mathrm{pp}}}}$ (i.e. the mean ${S_{{\mathrm{pp}}}}$ value across the three responding recordings) of the four channels during this experiment were summarized in table 2.

**Table 2.** Overview of the recording outputs’ parameters for four selected channels of the array 1.

	Ch1	Ch2	Ch3	Ch4
${\sigma _{{\mathrm{baseline}}}}$	$0.03370{\text{ mV}}$	$0.03310{\text{ mV}}$	$0.04785{\text{ mV}}$	$0.03583{\text{ mV}}$
${\overline {{\mathrm{SNR}}} _{{\mathrm{dB}}}}$	$37.66{\text{ dB}}$	$36.34{\text{ dB}}$	$36.01{\text{ dB}}$	$35.21{\text{ dB}}$
${\bar S_{{\mathrm{pp}}}}$	$ - 1.97{\text{ mV}}\,{{\mathrm{g}}^{{\mathrm{-1}}}}$	$ - 1.83{\text{ mV}}\,{{\mathrm{g}}^{{\mathrm{-1}}}}$	$ - 2.41{\text{ mV}}\,{{\mathrm{g}}^{{\mathrm{-1}}}}$	$ - 1.69{\text{ mV}}\,{{\mathrm{g}}^{{\mathrm{-1}}}}$

Overall, the results confirm stable and reproducible recording behavior across the four selected OECT channels. Under the baseline condition, all channels showed a stable signal level with minimal drift and low noise, providing a reliable reference for subsequent measurements. Under the active condition, the ${{\Delta }}{V_{{\mathrm{OUT}}}}$ traces from all channels followed the expected inverse polarity compared to ${{\Delta }}F$, consistent with the depletion-mode operation of the OECTs. The channels also exhibited comparable $\overline {{\mathrm{SN}}{{\mathrm{R}}_{{\mathrm{dB}}}}} $ values across the three responding recordings, while minor differences in ${\bar S_{{\mathrm{pp}}}}$ were observed. The overall recording behavior was consistent across channels. These findings demonstrate low inter-device variability within the array and further confirm the signal-recording performance of the portable system.

#### 3.2.2. Repeatability of recording

**Figure 9.**
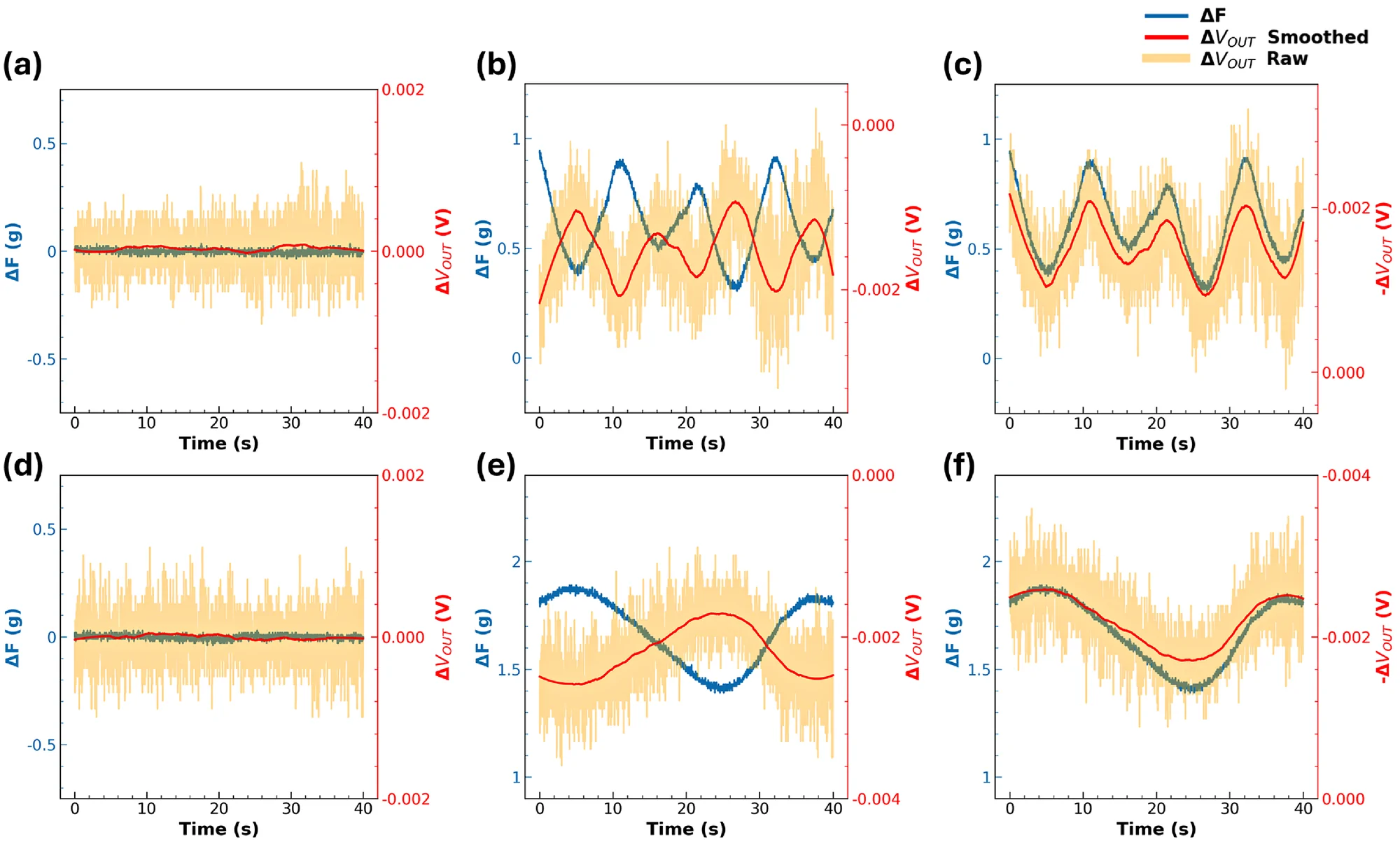
Simultaneous force-transducer and portable system recordings of selected channels from two independent OECT arrays in separate tests. Correspond to selected channel on OECT array 1 measured on tissue 1 (a) ${{\Delta }}{V_{{\mathrm{OUT}}}}$ and output force curves during the baseline period recording. (b) ${{\Delta }}{V_{{\mathrm{OUT}}}}$ and output force curves during the maximal contraction period. (c) The same Maximum spontaneously contractions state recording, system recording plotted with an inverted ${{\Delta }}{V_{{\mathrm{OUT}}}}$ axis. Correspond to selected channel on OECT array 2 measured on tissue 2. (d) Baseline state recording. (e) Maximal contraction period recording. (f) The same Maximum spontaneously contractions state recording, system recording plotted with the inverted ${{\Delta }}{V_{{\mathrm{OUT}}}}$ curve during maximal contraction period against the force curve.

To evaluate recording repeatability across different experiments, the results obtained from array 1 recording on tissue strip 1 (Experiment 1) were compared to those from array 2 recording on tissue strip 2 (Experiment 2). The selected channels ${{\Delta }}{V_{{\mathrm{OUT}}}}$ traces from both experiments were compared in figure 9 against the force sensor output. The recordings during the baseline period from the two experiments are shown in figures 9(a) and (d). Both arrays exhibited stable ${{\Delta }}{V_{{\mathrm{OUT}}}}$ signals during this period, with a ${\sigma _{{\mathrm{baseline}}}}$ value of 0.03370 mV for Ch1 of array 1 and 0.03252 mV for Ch1 of array 2.

The recording results during the period of maximal contraction are shown in figures 9(b) and (e). For Ch1 of array 1, the ${{\Delta }}{V_{{\mathrm{OUT}}}}$ trace followed the inverse polarity of the force transducer output, ${{\Delta }}F$, and yielded an ${\mathrm{SN}}{{\mathrm{R}}_{{\mathrm{dB}}}}$ of 35.81 dB and an ${S_{{\mathrm{pp}}}}$ of −1.93 mV g^−1^. In comparison, Ch1 of array 2, recorded under conditions with less frequent contractions in tissue strip 2, showed similar recording performance, with an ${\mathrm{SN}}{{\mathrm{R}}_{{\mathrm{dB}}}}$ of 38.11 dB and an ${S_{{\mathrm{pp}}}}$ of −1.89 mV g^−1^. Figures 9(c) and (f) present the same data as figures 9(b) and (e), respectively, but with the ${{\Delta }}{V_{{\mathrm{OUT}}}}$ axis inverted (-${{\Delta }}{V_{{\mathrm{OUT}}}}$) to facilitate comparison between the tissue contraction ${{\Delta }}F$ signal and the corresponding ${{\Delta }}{V_{{\mathrm{OUT}}}}$ from the portable system. The peaks and dips showed strong agreement in both cases, indicating that the response time of the portable system was sufficient to capture the low-frequency contractile activity of the bladder tissue strip. The ${\sigma _{{\mathrm{baseline}}}}$, ${\mathrm{SN}}{{\mathrm{R}}_{{\mathrm{dB}}}}$, and ${S_{{\mathrm{pp}}}}$ values of the selected channels from the two arrays were summarized in table 3. These results support good inter-sample repeatability of the arrays’ performance.

**Table 3.** Overview of recording outputs’ parameters for one selected OECT of arrays 1 and 2.

	Ch1 of array 1	Ch1 of array 2
${\sigma _{{\mathrm{baseline}}}}$	$0.03370{\text{ mV}}$	$0.03252{\text{ mV}}$
${\mathrm{SN}}{{\mathrm{R}}_{{\mathrm{dB}}}}$	$35.81{\text{ dB}}$	$38.11{\text{ }}dB$
${S_{{\mathrm{pp}}}}$	$ - 1.93{\text{ mV}}\,{{\mathrm{g}}^{{\mathrm{-1}}}}$	$ - 1.89{\text{ mV}}\,{{\mathrm{g}}^{{\mathrm{-1}}}}$

#### 3.2.3. Stability of recording

**Figure 10.**
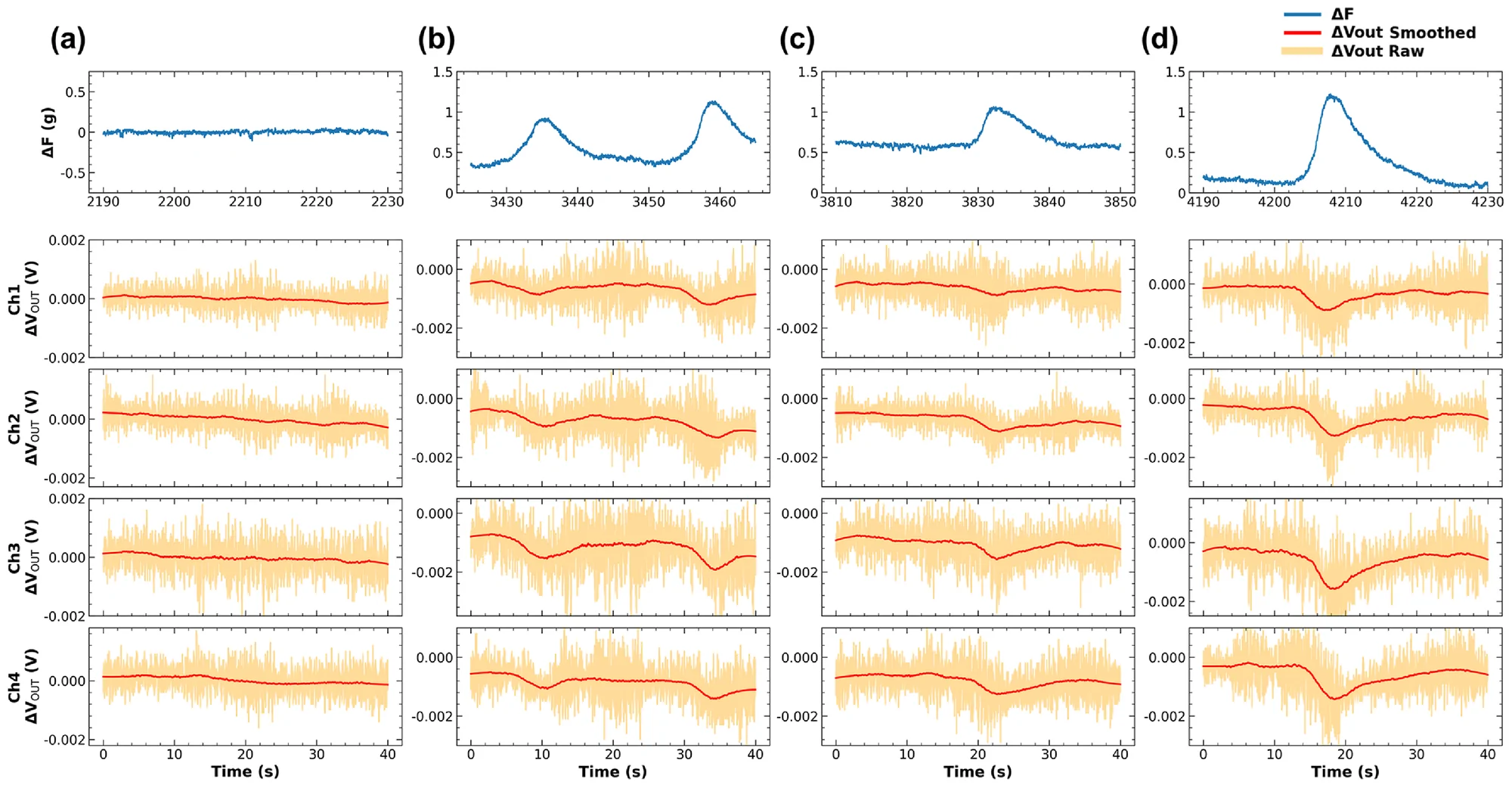
Simultaneous force transducer output and multi-channel recording four weeks after fabrication from OECT array 1 on tissue strip 3. (a) Baseline period recording. (b–d) Maximal contraction period recordings during three separate 40 s-blocks.

Recording stability was evaluated by repeating measurements on the same four channels of OECT array 1 on tissue strip 3 after a four-week interval (Experiment 3) and comparing them to the earlier dataset (Experiment 1). The recording results of Experiment 3 are shown in figure 10.

The ${{\Delta }}F$ from the force transducer remained nearly flat during the baseline recording period (figure 10(a)), confirming negligible contractile activity. The performance of the four channels was degraded, with ${\sigma _{{\mathrm{baseline}}}}$ increasing from 0.03310–0.04785 mV in Experiment 1–0.08760–0.1363 mV after four weeks, corresponding to percentage increases of 159.9%, 311.8%, 112.5%, and 214.3% for Ch1 to Ch4, respectively. Moreover, a gradual downward drift of approximately 0.3–0.6 mV was observed in Ch2 to Ch4 during the 40 s baseline period, indicating degradation of baseline stability.

In figure 10(b), the ${{\Delta }}F$ trace shows two contraction cycles, and two corresponding dips are observed in each channel’s ${{\Delta }}{V_{{\mathrm{OUT}}}}$ trace, with ${\mathrm{SN}}{{\mathrm{R}}_{{\mathrm{db}}}}$ values of 13.79–17.08 dB and ${S_{{\mathrm{pp}}}}$ values of −0.73 to −1.24 mV g^−1^. Similar to the result in Experiment 1, Ch3 exhibited the largest ${S_{{\mathrm{pp}}}}$ of −1.24 mV g^−1^. In figure 10(c), a larger magnitude for the contraction pattern is observed in the ${{\Delta }}{V_{{\mathrm{OUT}}}}$ trace, and all four channels display similar response patterns with $SN{R_{dB}}$ values of 14.58–18.09 dB and ${S_{{\mathrm{pp}}}}$ values of −0.81 to −1.41 mV g^−1^. ${{\Delta }}F$ in figure 10(d) shows the largest contraction pattern among the three recordings. All the channels’ output displayed ${{\Delta }}{V_{{\mathrm{OUT}}}}$ traces that clearly tracked the force signal, with ${\mathrm{SN}}{{\mathrm{R}}_{{\mathrm{dB}}}}$ values of 17.15–21.54 dB and ${S_{{\mathrm{pp}}}}$ values of −0.98 to −1.46 mV g^−1^. Table 4 summarizes the quantitative parameters. $\overline {{\mathrm{SN}}{{\mathrm{R}}_{{\mathrm{dB}}}}} $ decreased from 35.21–37.66 dB in Experiment 1–14.17–18.90 dB in Experiment 3, (a percentage decrease of −47.5% to −61.0%), and the ${\bar S_{{\mathrm{pp}}}}$ magnitude dropped from −1.69 to −2.41 mV g^−1^ in Experiment 1 to −0.84 to −1.37 mV g^−1^ in Experiment 3 (a percentage decrease of −32.5% to −57.4%). These percentage reductions in $\overline {{\mathrm{SN}}{{\mathrm{R}}_{{\mathrm{dB}}}}} $ and ${\bar S_{{\mathrm{pp}}}}$ values demonstrated the degradation of recording quality of the array after four weeks.

**Table 4.** Overview of the recording outputs’ parameters for four selected OECTs of the array 1 after 4 weeks (Experiment 3) and their comparison with Experiment 1 in terms of percentage increase/decrease.

	Ch1	Ch2	Ch3	Ch4
${\sigma _{{\mathrm{baseline}}}}$	$0.0876{\text{ mV}}$	$0.1363{\text{ mV}}$	$0.1017{\text{ mV}}$	$0.1126{\text{ mV}}$
${\sigma _{{\mathrm{baseline}}}}{\text{ increase}}$	+159.9%	+311.8%	+112.5%	+214.3%
${\overline {{\mathrm{SNR}}} _{{\mathrm{dB}}}}$	$15.92{\text{ dB}}$	$14.17{\text{ dB}}$	$18.90{\text{ dB}}$	$16.40{\text{ dB}}$
${\overline {{\mathrm{SNR}}} _{{\mathrm{dB}}}}{\text{ decrease}}$	−57.7%	−61.0%	−47.5%	−53.4%
${\bar S_{{\mathrm{pp}}}}$	$ - 0.84{\text{ mV}}\,{{\mathrm{g}}^{{\mathrm{-1}}}}$	$ - 1.06{\text{ mV}}\,{{\mathrm{g}}^{{\mathrm{-1}}}}$	$ - 1.37{\text{ mV}}\,{{\mathrm{g}}^{{\mathrm{-1}}}}$	$ - 1.14{\text{ mV}}\,{{\mathrm{g}}^{{\mathrm{-1}}}}$
${\bar S_{{\mathrm{pp}}}}{\text{ decrease}}$	−57.4%	−42.1%	−43.2%	−32.5%

This performance loss is likely associated with the PEDOT:PSS degradation caused by moisture exposure during the four-week period, which could affect the on-current and transconductance of the OECT devices, leading to a clear degradation in baseline stability and recording quality. Better device encapsulation and storage of the array in a controlled environment could slow down the PEDOT:PSS degradation. Despite the OECTs’ performance degradation over time, all four channels showed comparable performance and still produced reproducible responses corresponding to changes in force. Furthermore, all the channels exhibited larger ${{\Delta }}{V_{{\mathrm{OUT}}}}$ magnitudes when ${{\Delta }}F$ increased.

### 3.3. *Ex vivo* stimulation

**Figure 11.**
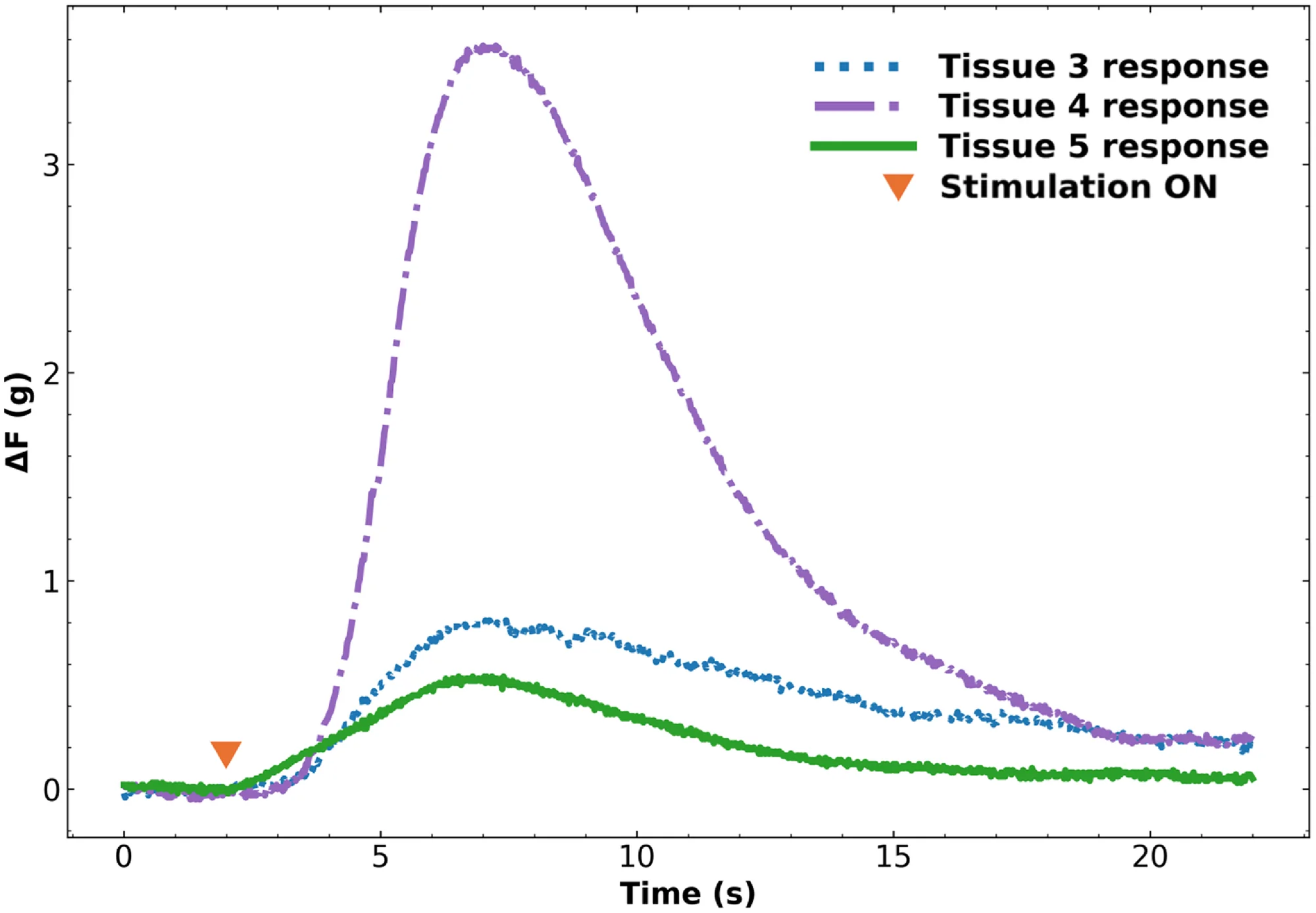
The portable system stimulation by the OECT arrays 1, 3, and 4 on 3 different tissue strips (3, 4, and 5), respectively. The force transducer output characteristics during each stimulation experiment.

Stimulation performance was evaluated by applying a 1 V, 50 Hz square-wave signal (with a duration of 5 s and a pulse width of 1 ms) from the portable system to the connected drain and source terminals of a selected OECT in Experiments 3, 4, and 5. The current *I*_G_ passed between the OECT channel and the Ag/AgCl electrode current sink, then stimulated the tissue underneath. The tissue activity due to the stimulation was then measured by the force sensor. Three different tissue strips were used for the stimulation experiments: Tissue strips 3, 4, and 5, which were stimulated by Ch5 of arrays 1, 3, and 4, respectively. To minimize interference from spontaneous tissue contractions, the stimulation experiment was conducted when the tissue was in the baseline period. The force transducer outputs from three separate tissue stimulation examinations are shown in figure 11, the start of the stimulation train is indicated by an orange triangle. All tissue strips exhibited a stimulation-evoked force increase shortly after stimulation onset, followed by a rise to a peak and gradual relaxation, confirming that the portable system reliably activated the bladder tissue. Under the same stimulation conditions, the peak amplitudes varied across specimens (3.63 g, 0.85 g, and 0.56 g), which likely reflects intrinsic biological differences among the tissue strips.

**Figure 12.**
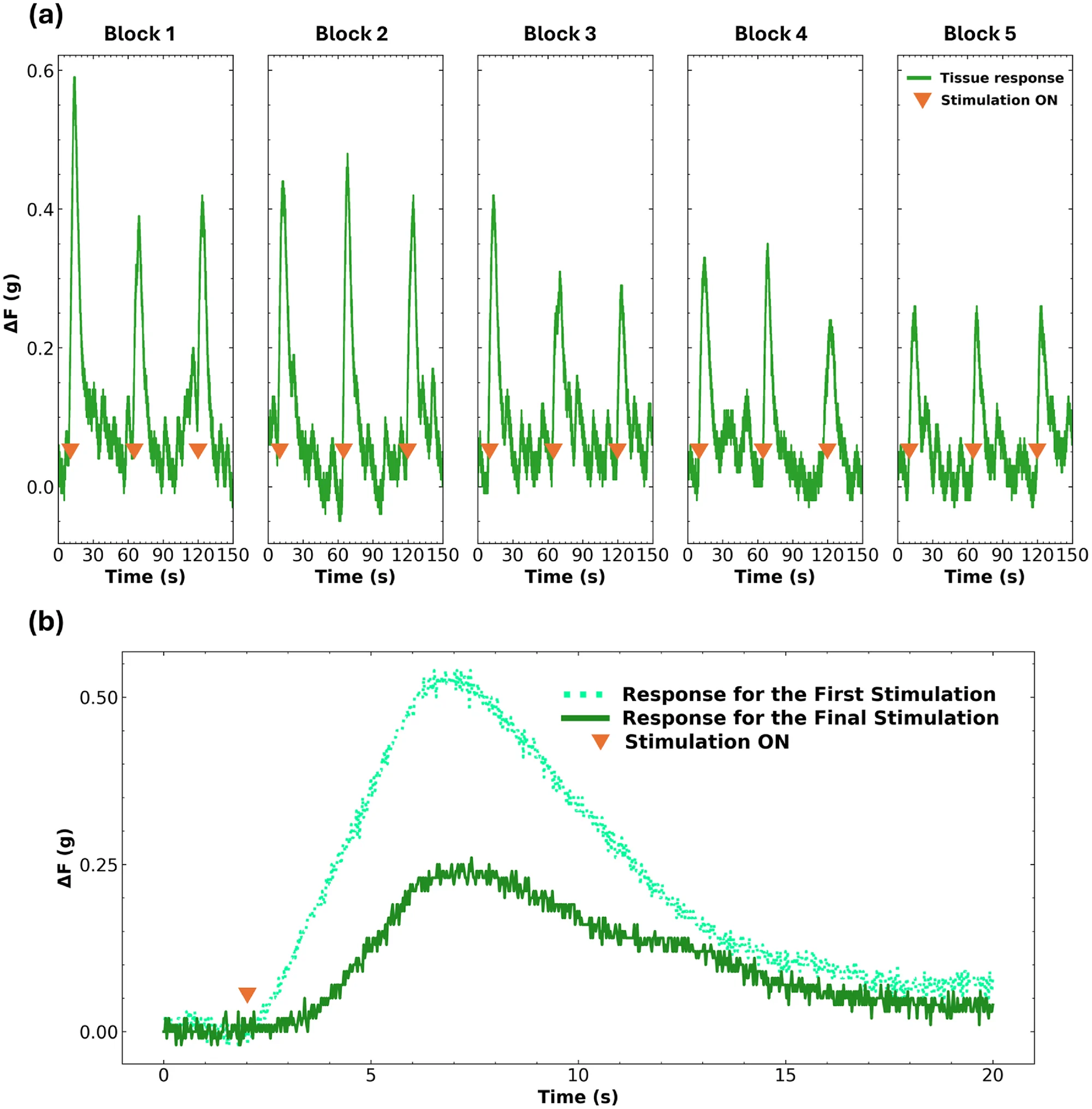
The portable system stimulation performance by the array 4 on tissue strip 5. (a) The force sensor output characteristics for the tissue in response to 5 continuous stimulation blocks, each containing three 5-second stimulations. (b) The magnified view of the force characteristics of the first and the final stimulations.

Repeatability of the stimulation performance was evaluated on tissue strip 5 using the Ch5 of array 4 during five stimulation blocks (Experiment 5). Each stimulation block lasted 150 s and contained three individual 5 s-stimulations separated by 50 s-idle intervals. Also, each block included a 10 s-delay at the beginning and a 25 s-delay at the end. An interval of approximately 180 s was introduced between consecutive blocks. The force transducer output for five stimulation blocks is displayed in figure 12(a). Each stimulation consistently evoked a transient contraction peak, demonstrating repeatable stimulation response during each block. A similar stimulation response was observed during five stimulation blocks with three contraction peaks in each block. Each contraction peak happened as soon as the 5 s-stimulation is ON. The onset of 5 s-stimulation is labeled with a triangle in figure 12(a). Figure 12(b) provides a magnified view of the force output in response to the first (Block 1) and the final (Block 5) 5 s-stimulation signals. The response amplitude decreased with repeated stimulation trains, which could indicate fatigue in the tissue strip during continuous stimulation. The peak value reduced from about 0.520 g in the first stimulation to around 0.238 g in the final stimulation, which is a reduction of 54.24%. The contraction and relaxation patterns were still clear, showing that stimulation was still effective despite the tissue fatigue.

## 4. Conclusion

This work develops a portable OECT-based platform as a bidirectional recording-stimulation interface for the bladder smooth muscle. The platform integrates multi-channel recording with electrical stimulation in a single, battery-powered system. The custom platform was able to conduct DC and AC characterization of OECTs and record OECTs’ outputs similar to a commercial parameter analyzer and apply stimulation waveforms during *in vitro* experiments. In *ex vivo* organ bath experiments, the platform exhibited low, stable baseline noise during inactive tissue periods and low-frequency output changes during spontaneous tissue activity similar to force transducer measurements. Consistent recording responses across different arrays and low variability among various OECTs within an array, indicate the capability of the platform for repeatable multi-channel recording. The platform consistently delivered electrical stimulation that triggered measurable bladder contractions in multiple tissue strips. Tissue stimulation responses varied across tissue specimens and attenuated with repeated stimulation, stressing the need to account for biological variability and fatigue. Overall, the recording-stimulation results position the presented platform as a practical foundation for closed-loop bladder control. Developing a recording-stimulation system in future will require further studies to establish a reliable relationship between the recorded signals and bladder state, including the urinary volume. This step should be followed by the development and validation of an appropriate control algorithm for the user to apply stimulation signals for voiding. Future chronic *in vivo* studies could record the OECT signal, conventional EMG signal and intravesical pressure simultaneously to establish a more complete relationship among electrical activation, tissue contraction and bladder function.

## Data Availability

All data that support the findings of this study are included within the article and the supplementary file. Supplementary data available at https://doi.org/10.1088/2058-8585/ae9bd1/data1.
